# Mitral annuloplasty with the interatrial groove-left atrial dome approach in a patient with Marfan syndrome

**DOI:** 10.1186/s13019-022-02031-1

**Published:** 2022-11-19

**Authors:** Fengjie Chen, Xiang Wang, Xianmian Zhuang, Hongwei Guo

**Affiliations:** 1 Department of Vascular Surgery, Fuwai Hospital Chinese Academy of Medical Sciences, Shenzhen, China; 2 Departments of Cardiovascular Surgery, Fuwai Hospital, National Center for Cardiovascular Diseases, Chinese Academy of Medical Sciences and Peking Union Medical College, Beijing, 100037 China

**Keywords:** Small left atrium, Mitral valve, The interatrial groove-left atrial dome approach

## Abstract

**Background:**

The choice of mitral valve surgical approach has always been a difficult problem in patients with small left atrium.

**Case presentation:**

We report a case of a patient with Marfan syndrome who underwent the David operation and mitral annuloplasty. The patient had a small left atrium, so we severed the superior vena cava and opened the interatrial groove and left atrial dome. This method allows for excellent exposure of the mitral valve and subvalvular apparatus, enabling a successful operation.

**Conclusion:**

The interatrial groove-left atrial dome approach provides an option for patients with a small left atrium undergoing mitral valve surgery.

## Introduction

For patients with a small left atrium (LA) undergoing mitral valve (MV) surgery, the choice of surgical route has always been challenging. We report a case of a patient with Marfan syndrome (MFS) and a small LA who underwent mitral annuloplasty through the interatrial groove-left atrial dome approach and the David operation. The operation achieved good early results.

## Case presentation

**Fig. 1:**
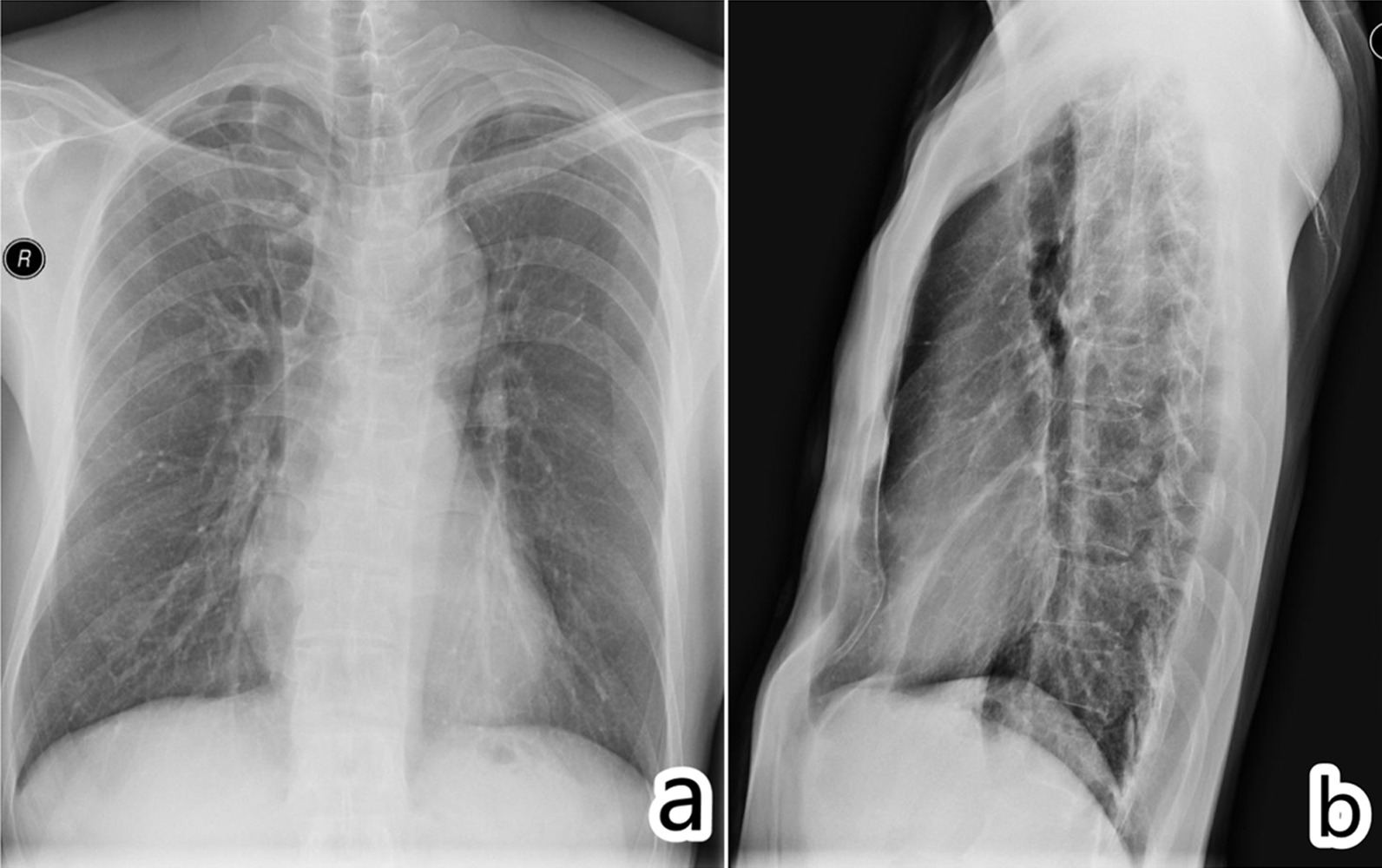
Chest radiographs. **a** Bilateral bullae, scoliosis; **b** Pectus excavatum

**Fig. 2:**
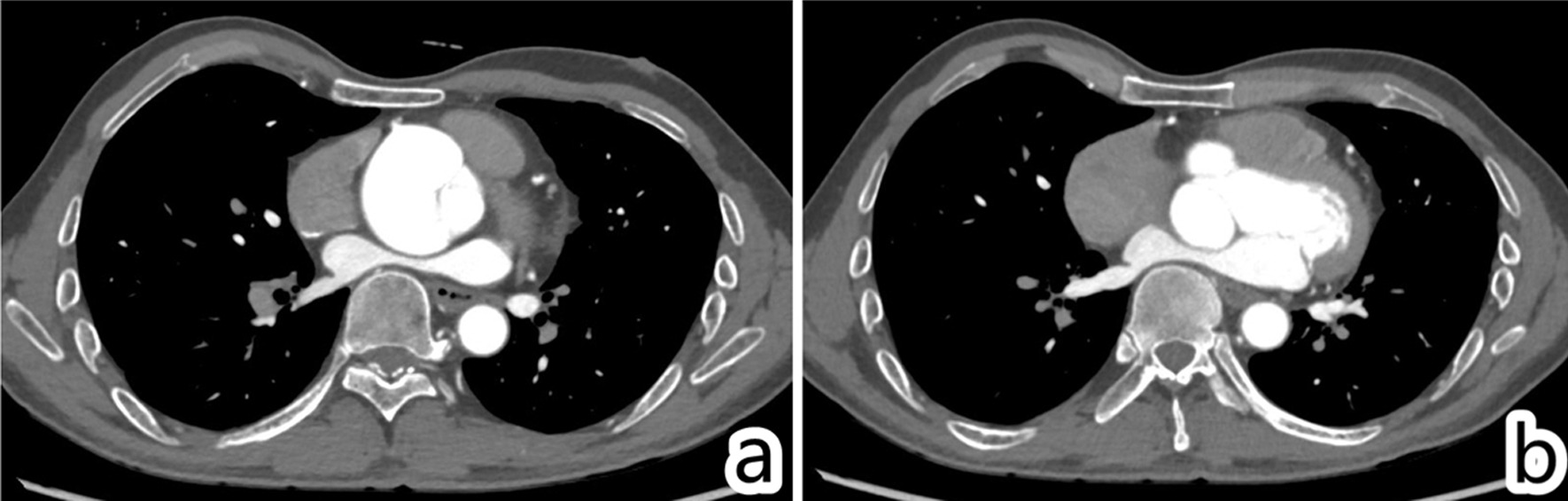
Chest computed tomography. **a** Aortic sinus aneurysm, approximately 54 mm in diameter; **b** the LA was compressed by the thoracic spine and aortic sinus aneurysm, with an anteroposterior diameter of only 20 mm

A 43-year-old male was admitted to our hospital with a 12-month history of shortness of breath and palpitations following physical exertion. Ultrasonography showed MV prolapse with mild to moderate regurgitation and mild aortic regurgitation, and the anteroposterior diameters of the LA, aortic valve annulus, ascending aorta and aortic sinus were 20 mm, 27 mm, 32 mm and 54 mm, respectively. Chest radiography showed bilateral bullae, scoliosis, and pectus excavatum (Fig. 1). Chest computed tomography showed an aortic root aneurysm, that the anteroposterior diameter of the thorax was flattened, and that the LA was compressed (Fig. 2). The electrocardiogram.

was normal. Considering the family history of MFS, genetic testing showed FBN1 mutations.

**Fig. 3:**
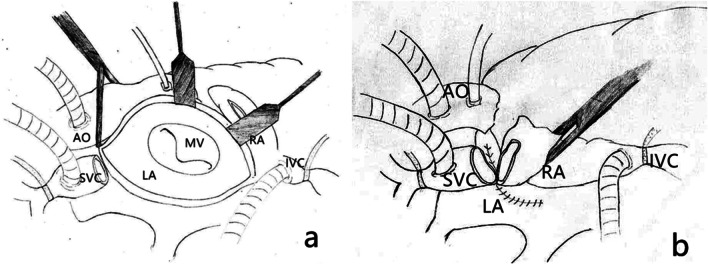
Surgical diagram. **a** Division of the SVC and opening of the interatrial groove-left atrial dome to expose the MV. **b** Continuous sutures to close the left atrium incision and anastomose the SVC

Surgery was performed through a median sternotomy. Cardiopulmonary bypass was established through the ascending aorta, superior vena cava (SVC), and inferior vena cava. The proximal end of the SVC was clamped and transected, taking care not to damage the sinus node. The interatrial groove and left atrial dome were exposed though an incision measuring approximately 4 cm (Fig. 3a). The MV and subvalvular apparatus were exposed, and a No. 32 Medtronic C-ring was implanted for mitral annuloplasty. The atrial incision and incision in the SVC were closed with running 5–0 Prolene sutures (Fig. 3b). The David operation was performed to treat the aortic root lesions. The cardiopulmonary bypass time was 334 min, and the cross-clamp time was 279 min. Intraoperative esophageal ultrasound showed minimal MV regurgitation and aortic valve regurgitation.

The postoperative hospital stay was 12 days. After 3 months of follow-up, the patient recovered well without complications such as arrhythmia and conduction block.

## Discussion and conclusions

The most commonly used approaches for MV surgery is the right atrium-atrial septum or interatrial groove approach, which can achieve satisfactory exposure in most cases; However, for special cases such as patients with a small LA, pericardial adhesions, or deep chest, it may be difficult to obtain satisfactory exposure with the conventional approach.

The patient in this case had a thoracic deformity. The LA was compressed by the thoracic spine and an aortic sinus tumor, which significantly reduced the anteroposterior diameter of the LA. It would have been difficult to obtain satisfactory MV exposure through the conventional approach. Some scholars have performed MV surgery through the left atrial dome approach and achieved.

good results [1, 2], and this approach is also suitable for patients with a small LA [3]. However, the patient had an aortic sinus aneurysm, which narrowed the gap between the aorta and the SVC. A simple left atrial dome incision would be too narrow to obtain satisfactory exposure. Therefore, we severed the SVC and combined the interatrial groove and left atrial dome approaches. This method can effectively increase exposure of the operative field and improve the surgical results, without complications such as arrhythmia or conduction block after the operation. Some authors believe that for patients with a small LA, division of the SVC for extended left atriotomy in MV operations is a viable approach [4, 5].

In summary, the interatrial groove-left atrial dome approach is an option for patients with a small LA for whom it is difficult to expose the MV.

## Data Availability

The datasets used and/or analysed during the current study are available from the corresponding author on reasonable request.

## Abbreviations

LA: Left atrium
MV: Mitral valve
MFS: Marfan syndrome
SVC: Superior vena cava
